# Proton beam therapy is a safe and effective treatment in elderly patients with esophageal squamous cell carcinoma

**DOI:** 10.1111/1759-7714.13524

**Published:** 2020-06-08

**Authors:** Takashi Ono, Hitoshi Wada, Hitoshi Ishikawa, Hiroyasu Tamamura, Sunao Tokumaru

**Affiliations:** ^1^ Department of Radiation Oncology QST Hospital Chiba Japan; ^2^ Department of Radiation Oncology Southern Tohoku Proton Therapy Center Fukushima Japan; ^3^ Department of Radiation Oncology and Proton Medical Research Center University of Tsukuba Ibaraki Japan; ^4^ Department of Radiation Oncology Proton Therapy Center, Fukui Prefectural Hospital Fukui‐ken Japan; ^5^ Department of Radiology Hyogo Ion Beam Medical Center Tatsuno City Japan

**Keywords:** Aged, aged 80 and over, esophageal neoplasms, proton therapy

## Abstract

**Background:**

There are many elderly patients with esophageal squamous cell carcinoma (ESCC). However, there are currently few articles regarding the clinical outcome following proton beam therapy in these patients. The purpose of this study was to evaluate the clinical results of proton beam therapy in elderly patients with ESCC.

**Methods:**

Between January 2009 and December 2013, patients aged ≥75 years who underwent proton beam therapy were examined using multi‐institutional data from Japan.

**Results:**

There were 38 inoperable patients (70.4%) and 16 operable patients (29.6%). More than 40% of patients had stage III/IV ESCC. The five‐year overall and cancer‐specific survival rates were 56.2% and 71.7%, respectively. Performance status was the only factor that significantly influenced overall survival during the multivariate analysis. The five‐year local control rate was 61.8%, and local recurrence occurred within 13 months in 82.4% of patients,. There was no grade 3 or higher toxicity, excluding three patients with grade 3 esophageal ulcers.

**Conclusions:**

In conclusion, proton beam therapy may become an alternative treatment with lower toxicity in elderly patients with ESCC, compared to surgery or conventional X‐ray radiotherapy. This includes inoperable patients.

**Key points:**

**Significant findings of the study:**

Proton beam therapy was a safe and effective treatment for elderly patients with esophageal squamous cell carcinoma (ESCC) including inoperable patients.

**What this study adds:**

Proton beam therapy may be a safer treatment choice for elderly patients with ESCC compared to conventional X‐ray radiotherapy.

## Introduction

Esophageal cancer (EC) is the seventh most common cancer, and its incidence rate is high in Eastern Asia.^1^ The incidence rate peaks among the elderly (between 60‐ and 70‐years‐old),^2^ and the number of elderly patients with EC is gradually increasing. Considering the proportional population changes predicted from 2005 to 2030, younger seniors aged 60–70 years will increase by 26%, and elderly patients aged 80–90 years will increase by 58%.^3^ In 2010, 31.7% of patients with EC in Japan were between the ages of 70 and 79 years, and 7.4% of patients with EC were 80 years or older, and squamous cell carcinoma (SCC) accounted for 90.5% of all cases of EC.^4^

If the EC is superficial, endoscopic treatment is recommended, even for elderly patients. However, if it is more advanced, surgery with or without chemotherapy and radical chemoradiotherapy (CRT) are the recommended treatment options.^2^ On the other hand, radical radiotherapy without chemotherapy may cure EC. However, adding chemotherapy leads to a much better overall survival, and CRT has become one of the treatment options considering the findings of the Radiation Therapy Oncology Group 85–01 trial.^5^

In Western countries, surgery with CRT is the standard treatment.^6^, ^7^ On the other hand, neoadjuvant chemotherapy followed by surgery is now the standard treatment as a result of the Japan Clinical Oncology Group (JCOG) 9907 trial^8^ in Japan. However, the JCOG 9907 trial involved only patients aged ≤75 years. Elderly patients were excluded from most clinical trials. This is because esophagectomy in elderly patients has been associated with increased in‐hospital mortality as well as increased pulmonary and cardiac complications.^9^ A prospective clinical trial involving radiotherapy without chemotherapy among patients aged ≥80 years with esophageal squamous cell carcinoma (ESCC) has been reported.^10^ However, even in this study, patients with T4 or lymph node metastasis were excluded.

Using the Bragg peak, proton beam therapy (PBT) can irradiate a targeted tumor sparing normal tissue irradiation compared to X‐ray therapy.^11^ Although no prospective study has compared the outcomes of radical PBT and radical X‐ray therapy in patients, Xi *et al*. reported that the overall survival (OS) rate of patients who underwent PBT was higher than that of patients who underwent intensity modulated radiation therapy.^12^ Therefore, PBT may improve clinical outcomes.

Regarding radical radiotherapy for elderly patients with ESCC, there have been several reports published including retrospective studies.^10^, ^13^, ^14^, ^15^, ^16^, ^17^ However, there have been no reports on PBT in elderly patients with ESCC. The objective of this study was to investigate the clinical outcome of elderly patients with ESCC.

## Methods

### Ethics statement

This retrospective multicenter study was approved by the ethics committees of all four institutions. The study was conducted in accordance with the Declaration of Helsinki.

### Patients

We retrospectively investigated the clinical results of elderly patients who received PBT using data from four PBT centers between January 2009 and December 2013 in Japan. This study included patients aged ≥75 years with a pathologically confirmed diagnosis of ESCC prior to treatment. Patients with metastasis to distant organs, treatment history of other organ cancer within five years before PBT and prior treatments for ESCC were excluded. Patients who received prior X‐ray therapy to irradiate prophylactic lymph node areas (elective nodal irradiation [ENI]) were included as the PBT fields were not large enough to cover the ENI field in all centers.

The clinical stage of ESCC (Union for International Cancer Control eighth edition) was assessed in all patients using endoscopy, esophagram, computed tomography (CT), and positron emission tomography (PET) scans.

### Treatment

Treatment strategies, including total dosage and chemotherapy regimens, were determined by a patient specific conference in each center. Whether ENI was used, or was not used, was decided by the treatment physicians in each center after considering the stage of the cancer, age, and performance status of the patient. The ENI field was decided by considering the region of primary ESCC. Typically, the ENI field included the following: (i) between the bilateral supraclavicular lymph node area and the area around the celiac artery when the carcinoma was in the thoracic region; (ii) between the aortic arch and the perigastric area when the carcinoma was near the esophagogastric junction; and (iii) between the hyoid bone and the carina including the bilateral supraclavicular lymph node area, when the carcinoma was in the cervical region. In conclusion, each treatment physician made fine adjustments to the ENI fields considering each patient's unique condition. The gross tumor volume (GTV) included primary ESCC and lymph node metastasis based on endoscopic and radiographic imaging. The clinical target volume (CTV) was defined as GTV plus 2–5 cm for both cranially and caudally, and plus 0.5–2 cm for other directions considering microinvasion. The planning target volume was defined as the CTV plus 0.5–1 cm. Prior to treatment, more than one clip was placed using endoscopy in all four PBT centers. Daily X‐ray imaging was used for positioning the bone and the clips.

### Evaluation of toxicities

Toxicities due to treatment were investigated using the Common Terminology Criteria for Adverse Events version 4.0.^18^ The occurrence of esophageal ulcers, esophageal fistulas, pericardial effusions, pleural effusions, and pneumonitis was investigated.

### Statistical analysis

The reported PBT dose was defined as the physical dose (Gy) multiplied by the relative biological effectiveness (RBE) of the protons. The RBE values for the protons were set to 1.1. Because multiple dose fractionations (1.8–2.2 Gy [RBE]) were used in each center, the effects of radiation on ESCC were compared using the biological effective dose (BED). We used an alpha/beta ratio of 10 Gy to calculate BED on the basis of the linear‐quadratic model, which is as follows: BED Gy (RBE) = total dose × (1 + dose per fraction/10). The Statistical Package for the Social Sciences software (SPSS: version 22, SPSS Inc., Chicago, IL, USA) was used to perform statistical analyses. The OS time was calculated from the first day of treatment to the time of last follow‐up or death. The cancer‐specific survival time was calculated from the first day of treatment to the date of death due to ESCC or last follow‐up. Regarding local recurrence, the locations where there was high dose irradiation or areas of marginal irradiation were not differentiated. The local control (LC) time was calculated from the first day of treatment to the day of local recurrence or last follow‐up. The OS rate, cancer‐specific survival rate, and LC rate were estimated using the Kaplan‐Meier method. Univariate and multivariate Cox regression analyses were performed to investigate the risk factors of OS. Significant and significant tendency factors (*P* < 0.1) in the univariate analysis were included in the multivariate analysis. All *P*‐values were two‐sided, and *P*‐values <0.05 were considered statistically significant.

## Results

### Patients

The characteristics of 38 inoperable patients (70.4%) and 16 operable patients (29.6%) are shown in Table 1. Half of the patients were 75–79 years old, and the others were ≥ 80 years. The median follow‐up time was 47 months (range: 2–112 months). There were 20 (37.0%) patients with lymph node metastasis and 22 (40.7%) patients with stage III/IV lymph node metastasis. The median total dose of BED 10, including the ENI dose, was 82.7 Gy (RBE); (range: 72.0–90.8 Gy [RBE]). More than half of the patients did not undergo chemotherapy.

**Table 1:** Patient characteristics

Characteristics	Patients
Follow‐up time	
Median (range)	47 (2–112) months
Gender	
Male	48 (88.9%)
Female	6 (11.1%)
Age	
Median (range)	79.5 (75–91) years
Performance status	
0	15 (27.8%)
1	26 (48.1%)
2	12 (22.2%)
3	1 (1.9%)
T category ^†^	
T1	21 (38.9%)
T2	10 (18.5%)
T3	17 (31.5%)
T4	6 (11.1%)
N category ^†^	
N0	34 (63.0%)
N1	14 (25.9%)
N2	5 (9.2%)
N3	1 (1.9%)
Stage ^†^	
I	21 (38.9%)
II	11 (20.4%)
III	14 (25.9%)
IV	8 (14.8%)
Tumor location	
Cervical	6 (11.1%)
Thoracic	48 (88.9%)
Total dose including elective nodal irradiation (BED 10)	
Median (range)	82.7 (72.0–90.8) Gy (RBE)
Elective nodal irradiation	
None	24 (44.4%)
Using proton beam therapy	7 (13.0%)
Using X‐ray therapy	23 (42.6%)
Area of elective nodal irradiation (*n* = 30)	
Supraclavicular area around the celiac artery	19 (63.3%)
Hyoid bone‐supraclavicular‐carina	9 (30%)
Aortic arch‐perigastric area	2 (6.7%)
Chemotherapy	
Cisplatin and 5‐fluorouracil	13 (24.1%)
Nedaplatin and 5‐fluorouracil	3 (5.5%)
Tegafur, gimeracil, and oteracil‐potassium	7 (13.0%)
None	31 (57.4%)

^†^ Numbers correspond to the tumor‐node‐metastasis system of classification (Union for International Cancer Control) eighth edition.

BED, biological effective dose; RBE, relative biological effectiveness.

### Survival

**Figure 1:**
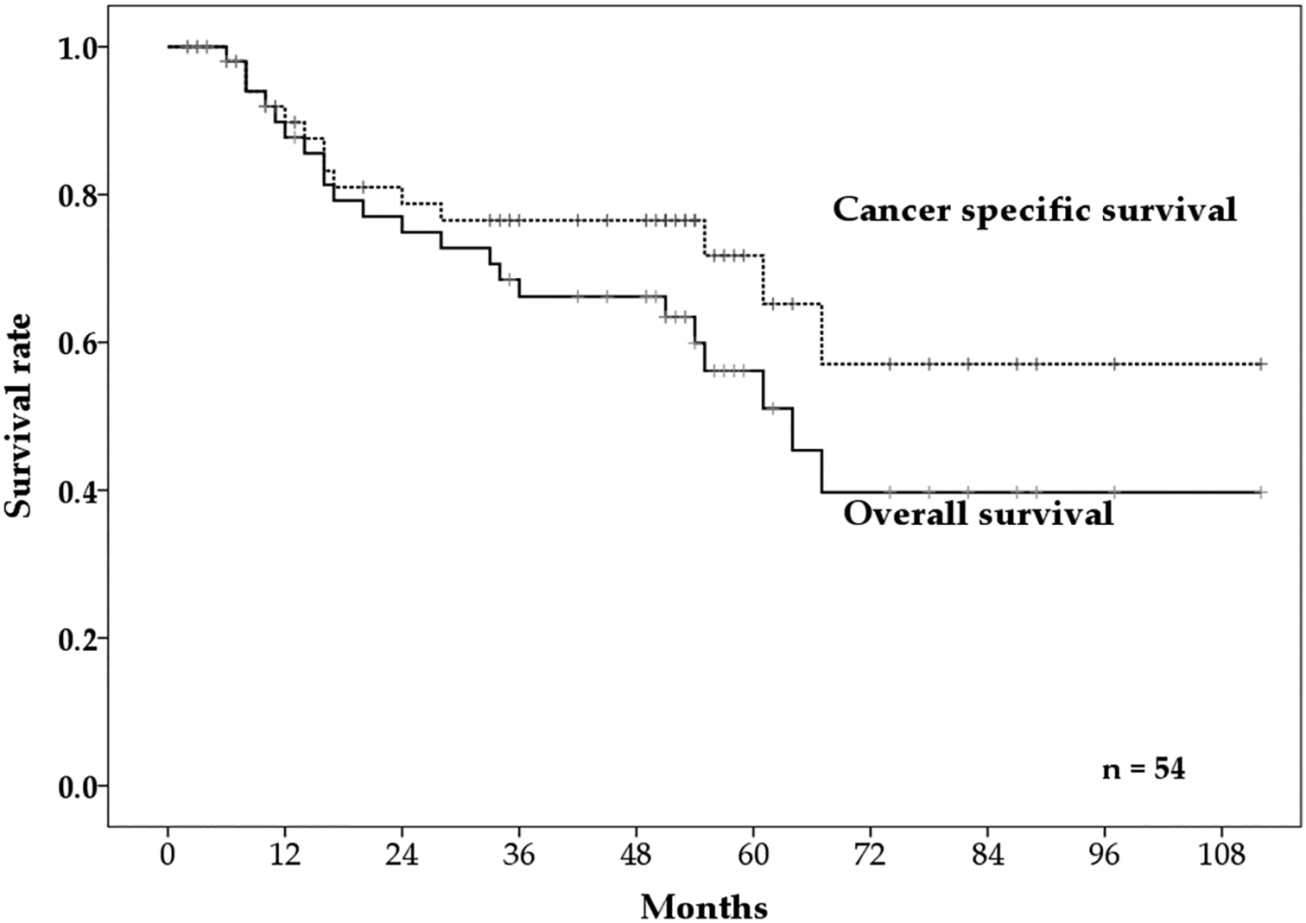
The two‐, three‐, and five‐year overall survival (OS) rate and three‐ and five‐year cancer‐specific survival rates.

**Figure 2:**
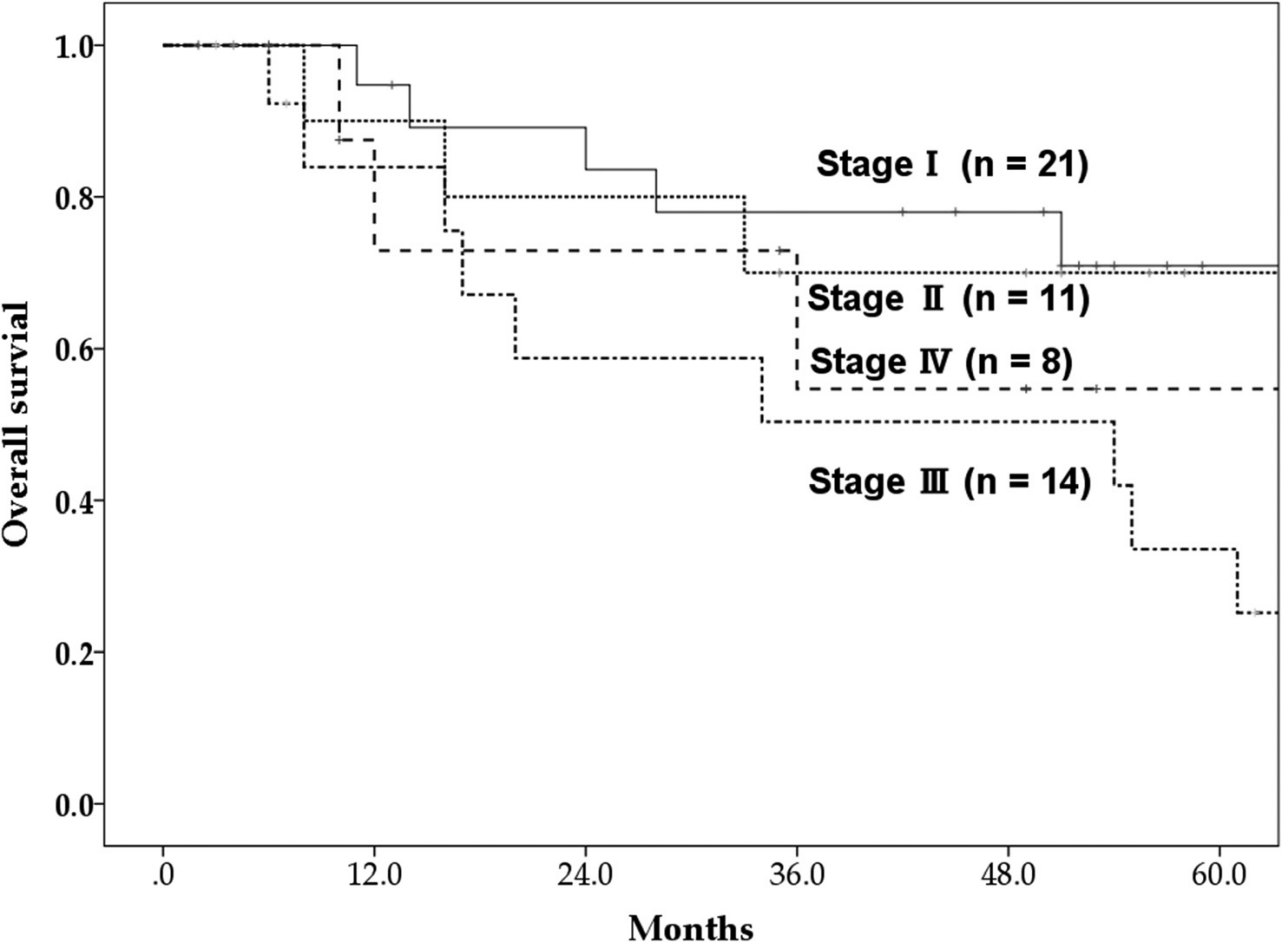
The five‐year overall survival rates of stage I–IV patients.

A total of 20 patients died. Among these patients, 12 died from primary cancer and the remaining eight died from other causes (four patients developed new cancers after PBT). The two‐, three‐, and five‐year OS rates were 74.9% (95% confidence interval [CI]: 62.4%–87.4%), 66.2% (95% CI: 52.5%–79.9%), and 56.2% (95% CI: 40.5%–72.0%), respectively (Fig 1). The median survival time was 64.0 months (95% CI: 50.5 months–77.5 months). The five‐year OS rates based on cancer stages I, II, III, and IV were 70.9%, 70.0%, 33.6%, and 54.7%, respectively (Fig 2). The three‐ and five‐year cancer‐specific survival rates were 76.5% (95% CI: 64.2%–88.8%) and 71.7% (95% CI: 57.0%–86.4%), respectively (Fig 1). A higher performance status was the only factor that was found to significantly influence the OS rate during the univariate analysis and, subsequently, during the multivariate analysis (Table 2).

**Table 2:** Univariate and multivariate analysis for overall survival

Factor	Comparison	Univariate analysis		Multivariate analysis	
		HR (95% CI)	*P‐*value	HR (95% CI)	*P‐*value
Age	75–79 vs. ≥80	0.61 (0.26–1.42)	0.254	‐	‐
Gender	Women vs. men	1.37 (0.80–2.36)	0.251	‐	‐
Performance status	Continuous	2.10 (1.26–3.51)	0.005 *	1.97 (1.04–3.74)	0.039*
Operability	Operable *vs*. inoperable	0.75 (0.47–1.20)	0.234	‐	‐
T category	Continuous	1.44 (0.96–2.16)	0.080	0.92 (0.53–1.58)	0.749
N category	Continuous	1.54 (0.98–2.42)	0.061	1.19 (0.41–3.46)	0.066
M1 lymph node metastasis	No or Yes	0.046 (0–720.09)	0.532	‐	‐
Total dose (BED 10)	<82.7 Gy (RBE) vs. ≥82.7Gy (RBE)	2.17 (0.88–5.34)	0.091	2.17 (0.88–5.34)	0.754
Elective nodal irradiation	No vs. Yes	0.87 (0.38–2.02)	0.750	‐	‐
Chemotherapy	No vs. Yes	0.468 (0.19–1.13)	0.092	0.68 (0.26–1.80)	0.435

BED, biological effective dose; CI, confidential interval; HR, hazard ratio; OS, overall survival; RBE, relative biological effectiveness. * *P*‐value <0.05

### Failure patterns

**Figure 3:**
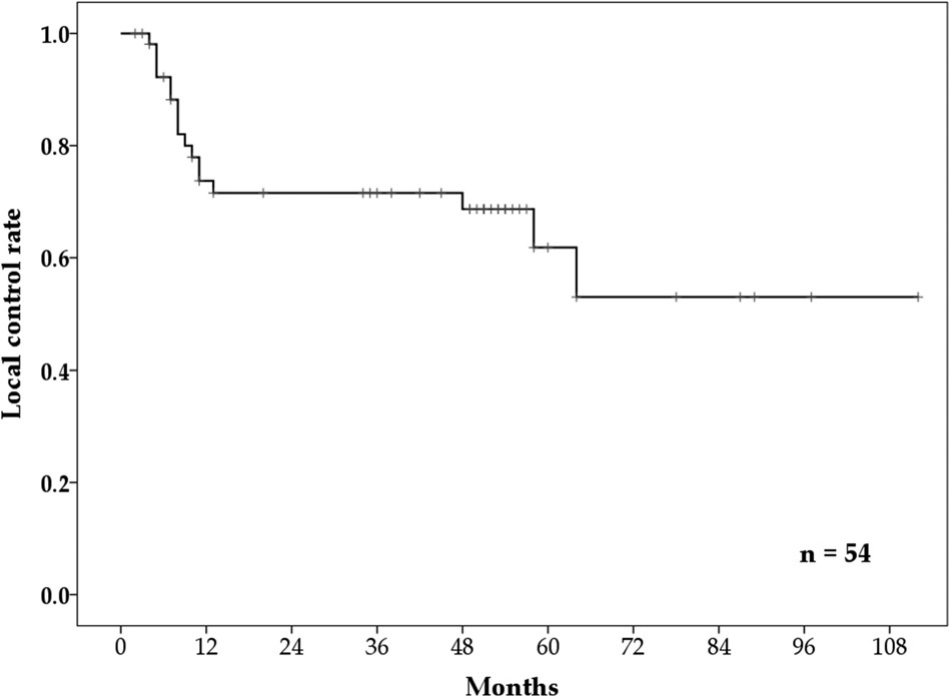
**The** three‐ and five‐year local control (LC) rates.

There were 17 patients who presented with local recurrence. Recurrence occurred in 14 of 17 patients (82%) within 13 months. In‐ and out‐field recurrence occurred in the lymph nodes of one patient, while distant metastases occurred in three patients. The three‐ and five‐year LC rates were 71.5% (95% CI: 58.6%–84.4%) and 61.8% (95% CI: 44.4%–79.2%), respectively (Fig 3). The five‐year LC rates based on cancer T categories one, two, three, and four were 72.0%, 77.8%, 45.1%, and 44.4%, respectively.

### Toxicities

There was no grade 3 or higher cardiopulmonary toxicity after PBT (Table 3). Four of six patients with grade 2 pericardial effusion received ENI (two patients underwent X‐ray therapy and two patients underwent PBT), and all patients with grade 2 pneumonitis received ENI using X‐ray. Regarding esophageal toxicity, there was no esophageal fistula, although three patients had grade 3 esophageal ulcers. No treatment‐related deaths occurred.

**Table 3:** **Patient** toxicities

Toxicities	Grade 0/1	Grade 2	Grade 3	Grade 4/5
Esophageal ulcer	34 (63.0%)	14 (31.4%)	3 (5.6%)	0
Esophageal fistula	54 (100%)	0	0	0
Pericardial effusion	48 (88.9%)	6 (11.1%)	0	0
Pleural effusion	51 (94.4%)	3 (5.6%)	0	0
Pneumonitis	52 (96.3%)	2 (3.7%)	0	0

## Discussion

To the best of our knowledge, the present study is the first study to provide data on PBT in elderly patients with ESCC.

If elderly patients with ESCC have a good performance status, surgery is considered the main treatment option. However, a systematic review by Markar *et al*.^9^ showed that in‐hospital mortality was approximately twice as high for elderly patients as that seen in younger patients (7.83% vs. 4.21%). They also reported that the five‐year OS rate decreased and cardiopulmonary complications associated with esophagectomy increased in elderly patients compared to younger patients. Yoshida *et al*. also reported that the risk of surgery‐related mortality increased as patients aged.^19^ In the aforementioned systematic review, the five‐year OS rate among elderly patients was 21.23%, which is low compared to the OS rate in the present study. Moreover, functional and cognitive impairment, depression, and social isolation were prevalent in elderly patients with EC and associated with worse health outcomes according to the systematic review by Deudekom *et al*.^20^ Therefore, surgery for elderly patients is a high risk. In fact, the number of elderly patients who underwent surgery dramatically reduced with age.^21^ In the present study, we observed a good OS rate with no severe toxicity, even among inoperable patients. If patients with early stage EC did not receive any treatment, the five‐year OS was 10% or less.^22^ This natural history was exceptionally low, and PBT possibly yielded much better OS than noncurative therapy for inoperable patients. It may be safer than surgery, especially in elderly patients with coexisting diseases such as ischemic diseases or chronic pulmonary dysfunction.

Several patients undergo radical radiotherapy because they cannot undergo surgery for the treatment of EC. Table 4 shows the clinical outcomes of elderly patients with primary ESCC who underwent radical X‐ray therapy and PBT [10, 13–17]. We observed a high OS rate with a low number of severe cardiopulmonary toxicities. Indeed, some previous studies reported worse OS rates and a high number of severe toxicities due to 2‐D X‐ray treatment. However, even when compared to the outcomes of patients following treatment with 3‐D X‐ray therapy, the outcomes of patients in our study were better. This suggests that PBT may be a superior treatment option for elderly patients compared to X‐ray therapy. In fact, Xi *et al*. reported that PBT improved the OS rate among patients compared to X‐ray therapy.^12^

**Table 4:** Clinical outcomes of elderly patients with mainly esophageal squamous cell carcinoma (ESCC) who underwent radical X‐ray therapy and proton beam therapy

	Number of patients	Age	Stage	Two‐year OS	Three‐year OS	Five‐year OS	MST	Grade ≥ 3 cardiopulmonary toxicities
Kawashima *et al*.^10^	51	≥ 80	cT1‐3N0M0	53%	39%	‐	30 months	8%
Zhao *et al*.^13^	122	≥ 75	cstage II–III	‐	‐	‐	22 months	0 (only lung)
Ji *et al*.^14^	30	≥ 70	cT1‐4N0‐1M0	45.1%	‐	‐	24 months	3% (only lung)
Kawamoto *et al*.^15^	84	≥ 76	cT1‐4N0‐3M0‐1	‐	33%	13%	21 months	5% (only lung)
Suzuki *et al*.^16^	50	≥ 75	cT1‐4N0‐3M0‐1	53%	‐	‐	‐	18%
Jingu *et al*.^17^	185	≥ 80	cT1‐4N0‐1M0‐1	‐	52.6%	‐	43 months	10%
Present study	54	≥ 75	cT1‐4N0‐3M0‐1	74.9%	66.2%	56.2%	64 months	0

MST, median survival time; OS, overall survival.

Combining chemotherapy with radical radiotherapy during treatment for elderly patients may result in severe toxicity and a worsened performance status. In young patients, concurrent chemotherapy results in a much higher OS rate, as reported by the Radiation Therapy Oncology Group 85–01.^5^ Zhao *et al*. also reported that the OS rate among elderly patients who underwent CRT was higher than that of elderly patients who underwent X‐ray therapy alone, although those who underwent CRT experienced severe toxicity.^13^ However, Jingu *et al*. reported that the three‐year OS rate for CRT was not significantly better than that for radiotherapy alone (53.7% vs. 59.9%).^17^ Adding chemotherapy was not a prognostic factor for OS in the present study. This may be because elderly patients experience severe late toxicity due to the addition of chemotherapy. On the other hand, the Comprehensive Registry of Esophageal Cancer in Japan, including radical and palliative radiotherapy, reported that the OS rate for CRT was significantly better than radiotherapy alone in stages II and III.^23^ To determine whether adding chemotherapy is useful in elderly patients with ESCC, further investigation is required.

Severe late toxicities, including toxicities of the heart and lungs, are also a major problem with radical radiotherapy for EC. Frandsen *et al*. reported that the history of X‐ray radiotherapy for EC was a significant predictive factor of death from heart disease (hazard ratio = 1.46). Furthermore, they reported that increased age was also a risk factor (hazard ratio = 1.74).^24^ Moreover, there have been some reports that investigated the correlation between heart dose and prognostic factors. Cai *et al*. reported that a higher ratio of heart volume irradiated >5 Gy was one of the significant predictive factors of a worse five‐year OS (hazard ratio = 1.01).^25^ Xu *et al*. reported that >45% of heart volume irradiated >30 Gy was one of the significantly worse factors of OS (hazard ratio = 1.42). They also reported that a mean lung dose >10 Gy was a significantly worse factor of OS (hazard ratio = 1.36).^26^ These findings suggest that higher heart or lung doses lead to worse OS, even if the patient recovers from the EC. A randomized phase IIB trial by Lin *et al*. reported that PBT led to a significantly lower total toxicity burden than intensity‐modulated radiation therapy.^27^ As shown in Table 4, PBT also caused less severe cardiopulmonary toxicity in the present study. This may be because PBT spares the surrounding heart and lung tissues compared to X‐ray therapy.^28^ PBT may also lead to a better OS due to a reduction in severe late toxicities.

There were some limitations to this study. First, this was a retrospective study. Second, the analysis of prognostic factors was limited by the number of patients provided. However, there are no prospective or retrospective studies on PBT in elderly patients with ESCC. Therefore, this data is significant. Third, this was a very heterogeneous patient set, involving patients with a wide range of disease stages. However, many previous studies also involved patients with a wide range of disease stages. Although it is not clear whether the combination of early stage and advanced disease is justified, we consider this article to be beneficial. Fourth, the treatment methods used at the four PBT centers were not unified. However, the outlines of the treatment methods were similar and did not have a significant effect.

PBT may become one of the treatment choices with low toxicity for elderly patients with ESCC, including inoperable patients. Further investigation is essential in order to determine the optimum treatment for these patients.

## Disclosure

All authors declare no conflicts of interest in association with the present study.
